# Molecular characterization of pine embryogenesis: pursuing the role of a putative non-specific lipid-transfer protein

**DOI:** 10.1186/1753-6561-5-S7-P71

**Published:** 2011-09-13

**Authors:** Marta Simões, Andreia Rodrigues, José de Vega Bartol, Raissa Santos, Célia Miguel

**Affiliations:** 1 IBET / ITQB-UNL, Av. República (EAN), 2780-157 Oeiras, Portugal

## Background

Embryogenesis in Gymnosperms presents unique characteristics when compared to Angiosperm embryogenesis. However, little is known about the molecular regulation of embryo development in Gymnosperms because most studies have been conducted in Angiosperm model species. Due to the economic relevance of some forest species included in this group of plants, namely pines, efforts have been made worldwide for the establishment of efficient clonal propagation methods, of which somatic embryogenesis is an example. A deeper knowledge of the molecular players regulating zygotic embryo development in pines will likely contribute to a better understanding and control over somatic embryo development *in vitro*.

We have previously performed a global gene expression analysis during maritime pine (*Pinus pinaster*) embryo development [1] and have selected a set of transcripts for further characterization based on their expression profile. One of such transcripts (*PpAAI-LTSS1*) putatively encodes a protein of the Alpha-Amylase Inhibitors (AAI)/Lipid Transfer (LT)/Seed Storage (SS) protein family, non-specific lipid-transfer protein (nsLTP)-like subfamily.The AAI-LTSS family of proteins is unique to higher plants and includes cereal-type alpha-amylase inhibitors, lipid transfer proteins, seed storage proteins, and similar proteins. Proteins in this family are known to play important roles, in defending plants from insects and pathogens, lipid transport between intracellular membranes, nutrient storage, as well as in developmental processes. *PpAAI-LTSS1* is up regulated in zygotic embryos at a pre-cotyledonary stage of development [2]. The aim of this work was to characterize in detail the expression patterns of *PpAAI-LTSS* along embryo development and its upstream regulatory sequence.

## Materials and methods

Zygotic embryos were isolated from immature cones and separated into five groups according to the developmental stage/collection date. The embryo staging was based on the system of Pullman and Webb [3] as described by Gonçalves *et al*. [4]. In each collection date, one or several consecutive embryo developmental stages were pooled as follows: Day 0 (stages T0, T1 and T2); Day 5 (stages T3 and T4); Day 11 (stage T4B); Day 15 (stage T5) and Day 25 (stage T7). Quantitative real-time RT-PCR (RT-qPCR) was performed using a iQ5 Real-Time PCR detection system (BioRad) to quantify PpAAI-LTSS1 transcripts in each of the five embryo groups to confirm differential expression along embryo development and developmental stage showing maximum up-regulation. Cloning of the upstream genomic region putatively corresponding to the promoter region, as well as localization of this transcript within the embryo tissues using in situ hybridization, are also being pursued.

## Results

Confirming previous results using microarrays, *PpAAI-LTSS1* transcript showed a peak of expression in developmental stages corresponding to pre-cotyledonary and early cotyledonary embryos decreasing onwards until embryo maturation. Preliminary *in situ* localization results point to specific expression of this gene in suspensor tissues but additional experiments are still running to confirm these observations. In addition, a 900bp genomic DNA fragment that putatively belongs to the promoter region of this gene has been cloned. The analysis of this DNA sequence revealed the presence of several conserved promoter motifs and no significant homology to any sequence present in public databases has been found. Further experiments will allow us to isolate the complete promoter sequence to be used in functional analysis studies.

## Conclusion

The transcript *PpAAI-LTSS1* putatively coding for a protein of the Alpha-Amylase Inhibitors (AAI)/Lipid Transfer (LT)/Seed Storage (SS) protein family may play an important role during embryo development in maritime pine, specifically at the pre-cotyledonary/early cotyledonary stage, as supported by quantitative gene expression data. The ongoing experiments for cloning the promoter region and for localizing specific expression in embryo tissues will provide additional clues for functional characterization.
